# Emergency rectal cancer: clinical presentation and therapeutic options

**DOI:** 10.3389/fsurg.2026.1778350

**Published:** 2026-02-23

**Authors:** Wanda Luisa Rita Petz, Olivier Gié, Rosita Sortino, Piercarlo Saletti

**Affiliations:** 1 Department of Digestive Surgery, Gruppo Ospedaliero Moncucco, Lugano, Switzerland; 2 Department of Medical Oncology, Gruppo Ospedaliero Moncucco, Lugano, Switzerland

**Keywords:** bleeding, emergency, obstruction, perforation, rectal cancer

## Abstract

Up to 20% of rectal cancer patients experience complications that result in an emergency presentation at diagnosis, the more frequent scenarios being represented by large bowel obstruction or perforation and tumor bleeding. The treatment of emergency rectal cancer depends both on patients' clinical conditions and risk factors, and on tumor's characteristics, primarily its intra- or extraperitoneal location. This article will address the different clinical presentations and the corresponding available treatments, with a particular focus on surgical techniques and multimodal chemoradiotherapy. In addition, it will address the prognosis of emergency rectal cancers and discuss healthcare policy strategies aimed at minimizing its occurrence.

## 1 Background

Rectal cancer accounts for about one third of all new cases of colorectal cancer worldwide. The incidence is approximately 3.9 per 100.000 population yearly (1), and projections suggest that by 2030 the incidence will rise also in individuals under 50 (2).

Even though screening programs of colorectal cancer have become the standard of care in most modern countries and, where adopted, have significantly increased early diagnoses (3, 4), emergency colorectal cancer still represent approximately 20% of the cases (5).

The more frequent emergent presentations of rectal cancer are represented by large bowel obstruction, tumor bleeding and tumor or colonic diastatic perforation; other clinical scenarios are severe pain or more rare conditions such as infectious complications or retroperitoneal organs invasion, for example ureteral obstruction requiring ureteric stents or nephrostomies.

The choice of the optimal treatment for a patient with an emergency presentation of rectal cancer depends on several patient and disease-related factors, namely physiological stability, tumor location (intra or extraperitoneal) and resectability, and the immediate need for symptom control.

Whenever possible, a full diagnostic work-up with colonoscopy and biopsies, thoraco-abdominal computed tomography and pelvic magnetic resonance is necessary with the fundamental aim of obtaining an accurate disease staging, keeping in mind that upfront surgery is rarely the treatment of choice of locally advanced extraperitoneal rectal cancer (LARC) which conversely benefits from multimodal therapy that significantly improves oncologic outcomes (6).

Consequently, unless local staging clearly demonstrates that surgical resection is appropriate as the initial treatment strategy, this should be avoided whenever possible for an extraperitoneal rectal cancer, and a stepwise approach is preferable, enabling proper assessment and subsequent guideline-based management. This strategy reflects “damage-control oncology,” converting an emergency into a staged pathway suitable for delayed oncologic therapy (7, 8).

Achieving oncologic adequacy with complete mesorectal excision, negative margins, and proper lymphadenectomy, is foundational in extraperitoneal rectal cancer surgery. As Bill Heald already emphasized in the 1980s (9), the fundamental principles of oncologic rectal surgery rely on meticulous dissection in the optimal tissue planes; however, emergency settings challenge these principles because of inflammation, contamination, and physiological instability.

Emergency surgical approach to colorectal cancer has been associated with an increased risk of R1 resection (10); similarly, inflammation and distorted anatomy may impair mesorectal dissection, although an increased rate of CRM involvement compared with elective surgery has not been consistently demonstrated.

The inflammation process obscures anatomy and makes autonomic nerve preservation often difficult, while enhancing the risk of damaging surrounding structures like genitalia (11); even an extensive nodal dissection, although crucial for staging and therapy, may compromise physiological reserve and abbreviated lymphadenectomy may be necessary.

Besides difficulties in achieving oncologic adequacy, patients' clinical conditions may discourage the attempt of performing a rectal resection with total mesorectal excision, which is a long and complex procedure, clearly inadvisable in conditions of physiologic instability because of the increased risk of bleeding and prolonged operative time.

In these cases, emergency treatment should emphasize rapid source control. Oncologic principles remain valid but must be adapted to emergency constraints: survival and stabilization come first, and radicality may be safely achieved in subsequent staged procedures (12).

## 2 Different clinical scenarios

### 2.1 Obstruction

Patients with bowel obstruction caused by advanced rectal tumor present typically with abdominal pain and distension, absence of stool and gas transit, nausea and vomiting; depending on the onset of symptoms, the clinical profile may range from acute abdomen requiring immediate assistance to a less evident clinical scenario with more subtle symptoms.

When approaching a patient with large bowel obstruction it must be kept in mind that the cause must be considered malignant until proven otherwise (13).

The diagnosis is confirmed by radiograph showing bowel distension, CT scan to exactly locate the site of obstruction, pelvic MRI to stage the tumor and, if possible, colonoscopy to obtain a diagnostic biopsy.

A comprehensive laboratory framework is necessary to evaluate the clinical consequences of bowel obstruction that may have caused fluids and electrolytes imbalance or anemia, to assess the nutritional status of the patient (protein and albumin levels) as this may influence the surgical decision making and to accurately stage the disease providing baseline data [carcinoembriogenic (CEA) levels] (14).

The treatment of an obstructive rectal cancer depends primarily on tumor location (7, 8).

Intraperitoneal lesions may benefit from surgical resection, loop colostomy or endoscopic stents.

Surgical resection should always be attempted, with the aim of both solving the obstructive complication and treat the underlying disease; the choice between Hartmann procedure and resection with immediate anastomosis mainly depends on surgeon's expertise and evaluation of local status and patient's frailty (15).

A one-step procedure is obviously advantageous because it eliminates the need of a second operation and the risk of a permanent stoma (16).

However, Hartmann's procedure is the most common emergency curative option for obstructing upper rectal or rectosigmoid cancers because it avoids an anastomosis under hostile conditions (17).

A useful tool in decision making process on whether attempting a one-step procedure with immediate anastomosis is represented by indocyanine-green assessment of adequate colonic perfusion, which has demonstrated, especially in the setting of elective rectal resections, to lower anastomotic leak rates (18–20).

If patients' clinical conditions do not allow for an extensive surgical procedure, or in case of non resectable rectal tumor, a loop colostomy may alleviate symptoms; in cases of massive bowel distention where mobilizing the colon for a formal loop colostomy is hazardous or difficult, a transverse loop colostomy with minimal mobilization may represent a damage-control technique to prevent iatrogenic injury.

In presence of a metastatic or unresectable tumor, when systemic chemotherapy is previewed, endoscopic self expandible metallic stents (SEMS) can be positioned if local expertise is available (14, 21).

In comparison with colostomy, SEMS have the advantage of ameliorating quality of live avoiding the need of diversion of intestinal transit and are generally better tolerated (22).

SEMS have also been employed as a bridge-to-surgery strategy (23), with the aim of treating obstructive status, stabilize the patient and allowing the performance of a radical oncologic surgical treatment once physiological stability reestablished; advantages of this approach are the increased possibility of performing a minimally invasive rectal resection once the emergency has been treated (24), and the avoidance of stoma creation.

However, definitive data on the superiority of bridge to surgery strategy in comparison with immediate surgery are lacking, and stent complications such as migration, reocclusion and rectal perforation, have been descripted with an incidence up to 13% (17); moreover, precautions are rising concerning potential risk of microperforation leading to decremental oncologic outcomes (25).

A different scenario is represented by extraperitoneal rectal cancers, which should be approached with non-resection strategies, particularly diversion alone, in a stepwise approach.

A loop colostomy relieves obstruction, lowers intraluminal pressure and stabilizes physiology, allowing patients to benefit from the most indicated multimodal oncological strategy (7, 8).

In these cases, endoscopic stent placement is generally not recommended due to proven technical difficulties, limited duration of effectiveness and high incidence of chronic pain, stent migration and rectal tenesmus, which may compromise optimal management and quality of life (23, 26).

### 2.2 Bleeding

Rectal cancer is the cause of lower gastrointestinal bleeding (LGIB), defined as hematochezia or bright red blood per rectum originating from a colorectal source, in 2%–11% of the cases, whereas the most common cause is diverticular disease (27, 28).

The treatment of patients experiencing acute rectal bleeding caused by rectal cancer depends primarily on hemodynamic stability.

The recent revision of the American College of Gastroenterology Guidelines for the management of acute LGIB (29) recommends for hemodynamically stable patients with a significant LGIB needing hospitalization a restrictive blood transfusion strategy (hemoglobin threshold for transfusion of 7 g/dL) except in patients with severe cardiovascular comorbidities, the performance of a colonscopy only if not already done in the previous twelve months and the reversal of antiplatelet or anticoagulant therapy.

This latter can be maintained in hemodynamically stable patients with a low risk LGIB, which can be managed in an outpatient basis and do not require hospitalization.

Endoscopic therapeutic modalities to achieve hemostasis include clipping, band ligation, argon plasma coagulation (APC) and injection of diluted epinephrine, with often a combination of mechanical and thermal approaches (30, 31).

If bleeding recurs after initial intervention, repeated procedures should be considered, employing additional techniques if feasible.

In hemodynamically instable patients, after initial resuscitation with intravenous fluids and transfusions, a contrast-enhanced CT scan is recommended, followed, when positive, by transcatheter embolization or therapeutic colonoscopy depending on Centre's facilities and experience (29).

According to several observational studies, radiotherapy can achieve bleeding control in a relevant subset of patients. However, treatment effectiveness appears to vary according to intrinsic tumor characteristics as well as the total radiation dose delivered, and the fractionation scheme adopted. In palliative care, short-term hypofractionated regimens are preferred, as they provide rapid symptom relief, good tolerability and a reduced therapeutic burden. These protocols usually involve the administration of a total dose of between 20 and 30 Gy distributed in a few fractions, especially in patients with limited life expectancy (32–34).

Surgical resection may be warranted for carefully selected patients who experience persistent or recurrent bleeding when non-surgical options are either unsuitable or have failed. The primary objective of surgical intervention is to secure lasting hemostasis while ensuring appropriate standards of care. Depending on the patient's condition, procedures may range from radical (low anterior or abdominoperineal resection) to palliative resection: in these cases, a Hartman procedure is most frequently employed.

### 2.3 Perforation

Bowel perforation of an advanced rectal cancer occurs most frequently at the tumor level, and it is caused by infiltration and consequent disruption of bowel wall; clinical manifestation depends on tumor location: a perforated intraperitoneal rectal cancer will cause acute abdomen with peritonitis and will require immediate emergency surgery, while a perforated extraperitoneal rectal cancer could cause a pelvic abscess or a recto-vaginal or recto-vescical fistula.

Perforated tumors are more frequently well differentiated, as well differentiation is usually characterized by reduced angiogenesis and therefore with increased hypoxia and necrosis, resulting in an easier tissue perforation (35).

Less frequently, an obstructive rectal cancer could cause bowel perforation of a more proximal colonic segment; in most of the cases this happens on the caecum, where the maximal parietal tension is reached when the intraluminal pressure increases (Laplace's law: tension = pressure × radius).

The treatment of a patient with rectal cancer perforation depends on patient's clinical conditions and on tumor location (intra or extraperitoneal).

As mentioned, a patient with an intraperitoneal rectal or diastatic caecal perforation will be admitted with an acute abdomen; the diagnosis will be confirmed by radiograms and CT scan.

In case of physiological deterioration, damage control strategies frequently imply non oncologic Hartmann resection or creation of a loop colostomy, and the option of an open abdomen might be evaluated.

Oncologic resection is however recommended in stable patients with generalized peritonitis due to perforated intraperitoneal rectal cancer or proximal colonic diastatic perforation; in this latter case, resection of the tumoral and of the proximal perforation site should be accomplished simultaneously (7).

The choice between resection with primary anastomosis (with or without protective ileostomy) and Hartmann procedure depends on local factors and patients' characteristics.

In selected patients with localized contamination and limited comorbidities, segmental resection with primary anastomosis may be considered (8).

A perforated extraperitoneal rectal cancer presenting as a pelvic abscess or a fistula represents a condition requiring complex decision-making.

If clinical conditions are permissive, a delayed strategy is the best therapeutic option, allowing symptoms control and adequate oncologic treatment with neoadjuvant radio-chemotherapy and subsequent surgery if requested.

Broad-spectrum antibiotic therapy and evaluation for radiologic drainage is indicated for pelvic abscess, and colostomy featuring for recto-vaginal or recto-vescical fistula.

In rare cases, a distal rectal tumor perforated in the surrounding soft tissues of the pelvis can lead to a complex infectious complication such as the Fournier's gangrene, a serious and life-threatening condition which needs immediate surgical approach (36) with extensive debridment and eventually negative-pressure therapy.

### 2.4 Other clinical presentations

Emergency presentation of rectal cancer can rarely happen during neoadjuvant chemoradiation.

Radiotherapy-induced tissue ischemia can result in severe necrosis of tumoral tissue leading to tumor perforation (37); in the reported cases, perforation has been managed by conservative treatment (bowel rest and antibiotics) (38) or urgent fecal diversion (37), depending on patients' conditions.

Because of treatment-induced mucosal ischemia, an abnormal angiogenesis in the superficial layer of the lamina propria of the rectum can occur (39), leading to rectal bleeding rarely evolving as a life-threatening condition.

Moreover, the thickening of the rectal mucosa, with transmural infiltration of neutrophils and lymphoplasma cells and fibrosis into the bowel wall, often referred to as chronic proctitis, may progress to a clinical scenario of intestinal obstruction (40).

Side effects of radiotherapy are intuitively dose dependent and more frequent if total administered dose exceeds 60 Grays; patients' susceptibility is represented by underlying inflammatory conditions such as Chronic Inflammatory Bowel Diseases, HIV infection, diabetes, hypertension, atherosclerosis and collagen vascular diseases.

Some genetic predispositions have also been identified, such as ataxia-telangiectasia, Fanconi's anemia, and Nijmegen break- age syndrome (41).

## 3 Surgical approach to emergency rectal cancer

Emergency presentations of rectal cancer as obstruction, perforation, bleeding, or tumor-related sepsis, require rapid surgical procedure. Choosing between open, laparoscopic, and robotic approaches depends primarily on physiological stability, anatomical distortion, and available expertise.

Minimally invasive surgical techniques, such as laparoscopy and robotics, have become increasingly utilized in emergency rectal cancer cases (42). These approaches are particularly considered for patients who are hemodynamically stable or only mildly unstable, where the physiological conditions allow for the benefits of reduced postoperative pain, fewer wound-related complications, and quicker recovery times (43).

The selection of minimally invasive methods is closely tied to patient conditions, the absence of diffuse contamination or severe anatomical distortion such as bulky tumors or dense, impassable adhesions in a previously operated abdomen.

Despite expanding indications for minimally invasive surgery, the open surgical approach continues to serve as the standard of care in most true emergency situations, as it allows for rapid access and intervention, which are critical in managing life-threatening complications.

As such, while minimally invasive strategies can be beneficial in carefully selected cases, open surgery remains the preferred option when immediate control and broad exposure are required to ensure patient safety (44). Its drawbacks, including higher rates of wound infection, pulmonary complications, and prolonged recovery, are outweighed by the need for immediate, life-saving intervention.

In comparison with open surgery, laparoscopy offers superior pelvic visualization, which can facilitate assessment of tumor extent and the safe creation of a stoma. Its limitations arise in the setting of massively dilated bowel loops, phlegmon, or advanced inflammatory changes, where safe manipulation becomes challenging.

In addition, emergencies typically involve conditions incompatible with prolonged Trendelenburg position and pneumoperitoneum can worsen cardiopulmonary function in unstable patients (45). Conversion rates are higher than in elective surgery, reflecting the complexity of emergency pathology rather than technique failure (46).

Robotic surgical systems have been introduced to overcome technical limitations of laparoscopy, therefore expanding applications of minimally invasive surgery.

The high degree three-dimensional vision of the stable camera platform, the increased dexterity of endo-wristed instruments, tremor abolition, motion scaling, and the possibility of simultaneously utilise two energetic devices are characteristics of robotic platforms that can ameliorate surgical performance (47–49).

Despite the recent increase of the adoption of robotics in various surgery procedures (50) and the proven advantages of robotics in comparison with laparoscopy in terms of reduced conversion even in the emergency setting (42), main limitations to its use in emergency are the elevated cost of the platform, the need for an adequate training for the entire surgical team, together with anaesthesiologists and operative room nurses, and the robotic device availability 24 h and 7 days a week.

The same drawbacks of prolonged Trendelenburg and pneumoperitoneum already mentioned for laparoscopy in emergency are obviously valid also for the robotic approach to rectal cancer.

Therefore, the robotic approach remains marginal in emergencies and robotic surgery is still reserved for exceptional cases in stable patients during staffed hours (51).

Minimally invasive surgery can be effectively employed in the above mentioned highly effective hybrid strategies both in the initial phase such as initial bowel decompression with a stoma or laparoscopic control of sepsis, and in the following definitive oncologic resection (TME) either laparoscopic or robotic (7, 8, 52).

## 4 Multimodal treatment of rectal cancer

Once patients' conditions have been stabilized, the definitive treatment of an extraperitoneal LARC will entail a multimodal approach.

Multidisciplinary teams including colorectal cancer surgeons, medical oncologists, radiation oncologists, radiologists, gastroenterologists, pathologists and molecular biologists are of paramount importance to best treat patients with locally advanced rectal cancer (LARC), which includes stage cT3–4 and/or nodal positive disease.

In LARC, neoadjuvant strategies of preoperative chemo-radiotherapy have been evaluated to reduce the risk of local recurrence, downstage the tumour to facilitate radical surgery, increase pathological complete responses (CR) and reduce the risk of distant metastases impacting disease-free (DFS) and overall survival (OS).

Regimens avoiding radiotherapy have been proposed for cT2 nodal positive or cT3 tumors, and showed non-inferiority to standard chemoradiotherapy with respect to disease-free survival (53).

Selected patients who achieve a clinical CR after neoadjuvant treatment may be considered for active surveillance (the so-called watch-and-wait strategy) rather than surgery, enabling organ preservation and avoiding operative morbidity.

Long-course chemoradiotherapy (LCR) and short course radiotherapy (SCR), the latter more popular in northern Europe, have been the most used neoadjuvant strategies since many years (54, 55).

More recently, the total neoadjuvant therapy (TNT) approach emerged, typically recommended in stage II/III, mid or low rectal adenocarcinoma.

As adherence to systemic therapy is improved in preoperative than adjuvant setting, by early delivering of systemic therapy one of the potential benefits of TNT is the reduction of risk of distant metastases.

TNT can be conducted in two modalities, either as induction (systemic therapy followed by radiation therapy) or consolidation (upfront radiotherapy followed by chemotherapy), followed by evaluation for surgery.

Many trials have been designed in both consolidation (56–58) and induction sequence (59–61), with differences regarding the inclusion criteria, in particular local stage.

Overall, TNT is associated with increased pathological CR rate and improved DFS.

In support of this evidence, three recent meta-analyses showed that TNT improves pathological CR, DFS and OS in comparison with conventional neoadjuvant strategies (62–64).

Nevertheless, the optimal neoadjuvant treatment is not established yet, and TNT decision should be tailored based on locoregional stage and other risk factors such as mesorectal fascia involvement and extramural venous invasion.

Reported toxicity of TNT (notably neutropenia, peripheral neuropathy and gastrointestinal) varies among regimens and sequences.

In STELLAR trial, grade 3 adverse events were significantly higher in the TNT arm compared to standard CRT (58).

Conversely, in the RAPIDO (57) and UNICANCER-PRODIGE-23 (59) trials, there was no increased incidence of grade 3 adverse events vs. standard arm, and similar observations have emerged from two meta-analyses, the most recent reporting on modified TNT regimens (65, 66).

What about operative complications of TNT? Pelvic fibrosis can potentially affect sphincter preservation, radicality, lymph node yield and blood loss.

Timing of surgery is crucial for the surgical outcome. After TNT, in a retrospective trial, patients operated beyond 24 weeks experienced higher rates of reoperations (21% vs. 8%, *p* = 0.04) and severe complications (28% vs. 13%, *p* = 0.03) compared to those operated earlier; similarly, the risk of margin positivity was higher (17% vs. 3%, *p* < 0.01) in the first group (67). In the randomized phase II TIMING trial, patients received CRT followed by surgery (group 1) or CRT followed by two cycles of FOLFOX (group 2), four cycles of FOLFOX (group 3), six cycles of FOLFOX (group 4) before surgery. Although pelvic fibrosis was more pronounced in patients with longer interval between CRT and surgery, sphincter preservation, margin status, number of lymphnodes retrivied and blood loss were similar across all groups (68).

Surgical complications (anastomotic leak, abcess, abdominal bleeding, ileus and fistula) following TNT are superimposable to those observed with standard neoadjuvant CRT, as shown in meta-analyses and randomized trials (64, 66, 69, 70).

In the UNICANCER-PRODIGE-23 trial, post operative complication (29% vs. 31%, *p* = 0.66) and anastomotic leak or abcess (10% vs. 11%) were similar in the TNT arm compared to the standard arm. In a meta-analysis of SCR followed by consolidation chemotherapy vs. conventional neoadjuvant CRT, there were no significant differences (42% vs. 37%, *p* = 0.06) in terms of complications between the two strategies (71). In addition, no differences in the completeness of the mesorectal specimen has been reported (59). Of note, in the 5-year follow-up of the RAPIDO trial (72), a significant increase of breach of the mesorectum was reported in the TNT arm as compared to the standard arm (21% vs. 4%, *p* = 0.048).

Some factors can predict the risk to develop severe or fatal complications during TNT. First, patient-related factors such as advanced age (73, 74), visceral obesity (75), poor baseline nutritional and inflammatory status (low albumin, low prognostic nutritional index, elevated neutrophil-to-lymphocyte ratio) are linked to increased risk of complications and potentially worse survival (73). Tumor-related factors like can also negatively impact on treatment toxicity and outcome: among these are advanced locoregional stage, poor tumor regression grade and mutation in KRAS or BRAF genes, which are linked to a less frequent response to neoadjuvant therapy and are associated with higher recurrence and complication rates (76).

## 5 Prognosis

Even though colon and rectal cancer are well distinct entities with different biologic characteristics, treatment guidelines and prognosis, in most of the epidemiologic medical literature, including studies concerning the prognosis of cancer presenting in emergency, they are treated together with the appellation of *“colorectal cancer”*.

Consequently, data regarding emergency rectal cancer must be deducted from the wider group of emergency *colorectal* cancer.

Prognosis of colorectal cancer with emergent presentation has repeatedly been described as worse than that of cancer with elective presentation.

Obstruction represents a bad prognostic factor, with worse overall and disease-free survival for patients with obstructive stage III cancer when compared to patients with stage III non-obstructive tumors (77); an evocated explanation is that bowel walls distention caused by obstruction may lead to increased mucosal permeability and an easier release of factors that promote metastases.

Tumor perforation significantly worsens prognosis due to intraperitoneal or mesorectal tumor cell dissemination.

In all cases with emergency presentation, a curative resection is performed less frequently than in elective patients, postoperative morbidity and mortality are higher both in curative and in palliative resections, while overall and cancer-specific 5-years survival are poorer (78–82).

This is intuitively correlated to specific tumor characteristics frequently associated with emergent presentation and representing a risk factor for aggressive behavior, as shown in a recent systematic review with meta-analysis of more than nine hundred thousand patients: tumor size, advanced stage, T4, N positive and metastatic tumor, vascular, lymphatic and perineural invasion and poor differentiation at histology (83).

However, worse prognosis of emergent colorectal cancer has been highlighted independently from tumor stage, and emergency presentation constitutes itself a high-risk factor suggesting evaluation for adjuvant chemotherapy even for node-negative patients (77, 84, 85).

Patients' characteristics significantly associated to emergency colorectal cancer presentation have also been identified: these are advanced age (83, 86, 87), female sex (83, 86, 88), comorbidities, and all forms of social, economic, and personal disadvantage that can lead to a diminished propensity or reduced opportunity to engage with screening programs, and consequently to obtain an early diagnosis.

The association of advanced patients age and emergency colorectal cancer could be partially explained by the less awareness of screening availability, modalities and importance of older adults (>75 years) compared with younger patients (aged 65–74) as shown in a population-based survey published in 2008 (89).

Some studies have emphasized the poor compliance of women with screening programs that include a colonoscopy, which is perceived as painful and embarrassing, especially if performed by a male specialist (90–92).

Concerning comorbidities, it can be argued that patients with multiple concomitant illnesses are likely to focus their attention on their chronic health problems rather than pay attention to the presenting symptoms of colorectal cancer.

Among comorbidities, Wallace (86) identified dementia, cerebrovascular disease, hemi- and paraplegia as frequently present in patients with emergent colorectal cancer.

Socioeconomic deprivation, lower income, (88), lower use of primary care (93), being unmarried or widow (94), belonging to a ethnic minority (95) are, as mentioned, hallmarks of patients who are disinclined to attend to their own well-being, who fail to heed even the faintest symptoms that might herald a diagnosis of cancer or who simply face greater difficulties in accessing high-quality medical care.

Efforts must therefore be made to increase screening efforts in elderly patients, females, individuals with high comorbidities and different types of social disadvantage, with the aim of reducing those presenting in emergency and ameliorate prognosis.

A distinct scenario is represented by those patients who, despite having access to screening programs and visits with their general practitioner, experience delays in diagnosis and in specialist referral, as well as by those patients in whom the diagnostic delay is attributable to nonspecific symptoms.

A longitudinal study published in 2016 (96) showed that general practitioner consultation rate of patients presenting with emergency rectal cancer is increased in the year preceding the diagnosis, although often with non-typical symptoms; moreover, up to one fifth of them presented with “red flag” symptoms in the six to twelve months before diagnosis, representing therefore a subgroup in which the emergent presentation could have been avoided.

Major attention must hence be given to any patient consulting with increased frequency than usual, even with symptoms not immediately suggestive of cancer.

## 6 Conclusion

Emergency rectal cancer encompasses a wide spectrum of clinical presentations, with severity ranging up to life-threatening conditions.

Management should address both control of the acute condition and treatment of the underlying oncological disease. While surgical resection, when technically feasible and clinically safe, often represents the primary option for intraperitoneal tumors, a stepwise strategy should be favored for extraperitoneal tumors. In this setting, priority should be given to procedures that stabilize the patient and allow subsequent access to chemoradiotherapy, which represents the main curative option.

A multidisciplinary approach involving all key professionals required for accurate diagnostic assessment and optimal therapeutic planning (radiologists, anesthesiologists, gastroenterologists, surgeons, medical oncologists, and radiation oncologists) is essential to provide patients the appropriate treatment.
